# Dimethyl itaconate is effective in host-directed antimicrobial responses against mycobacterial infections through multifaceted innate immune pathways

**DOI:** 10.1186/s13578-023-00992-x

**Published:** 2023-03-08

**Authors:** Young Jae Kim, Eun-Jin Park, Sang-Hee Lee, Prashanta Silwal, Jin Kyung Kim, Jeong Seong Yang, Jake Whang, Jichan Jang, Jin-Man Kim, Eun-Kyeong Jo

**Affiliations:** 1 Department of Microbiology, Chungnam National University School of Medicine, Daejeon, South Korea; 2 Infection Control Convergence Research Center, Chungnam National University School of Medicine, Daejeon, South Korea; 3 Department of Medical Science, Chungnam National University School of Medicine, Daejeon, South Korea; 4 Brain Korea 21 FOUR Project for Medical Science, Chungnam National University School of Medicine, Daejeon, South Korea; 5 Center for Research Equipment, Korea Basic Science Institute, Cheongju, Chungbuk South Korea; 6 Department of Microbiology, Keimyung University School of Medicine, Daegu, South Korea; 7 Department of Research and Development, Korea Mycobacterium Resource Center (KMRC), The Korean Institute of Tuberculosis, Osong, 28158 South Korea; 8 Division of Life Science, Department of Bio & Medical Big Data (BK21 Four Program), Research Institute of Life Science, Gyeongsang National University, Jinju, 52828 South Korea; 9 Department of Pathology, Chungnam National University School of Medicine, Daejeon, South Korea

**Keywords:** Dimethyl itaconate, *Mycobacterium tuberculosis*, Nontuberculous mycobacteria, Innate immunity, Autophagy, Host-directed therapeutics, Antimicrobial responses

## Abstract

**Background:**

Itaconate, a crucial immunometabolite, plays a critical role in linking immune and metabolic functions to influence host defense and inflammation. Due to its polar structure, the esterified cell-permeable derivatives of itaconate are being developed to provide therapeutic opportunities in infectious and inflammatory diseases. Yet, it remains largely uncharacterized whether itaconate derivatives have potentials in promoting host-directed therapeutics (HDT) against mycobacterial infections. Here, we report dimethyl itaconate (DMI) as the promising candidate for HDT against both *Mycobacterium tuberculosis* (Mtb) and nontuberculous mycobacteria by orchestrating multiple innate immune programs.

**Results:**

DMI per se has low bactericidal activity against Mtb, *M. bovis* Bacillus Calmette–Guérin (BCG), and *M. avium* (Mav). However, DMI robustly activated intracellular elimination of multiple mycobacterial strains (Mtb, BCG, Mav, and even to multidrug-resistant Mtb) in macrophages and in vivo. DMI significantly suppressed the production of interleukin-6 and -10, whereas it enhanced autophagy and phagosomal maturation, during Mtb infection. DMI-mediated autophagy partly contributed to antimicrobial host defenses in macrophages. Moreover, DMI significantly downregulated the activation of signal transducer and activator of transcription 3 signaling during infection with Mtb, BCG, and Mav.

**Conclusion:**

Together, DMI has potent anti-mycobacterial activities in macrophages and in vivo through promoting multifaceted ways for innate host defenses. DMI may bring light to new candidate for HDT against Mtb and nontuberculous mycobacteria, both of which infections are often intractable with antibiotic resistance.

**Supplementary Information:**

The online version contains supplementary material available at 10.1186/s13578-023-00992-x.

## Background

*Mycobacterium tuberculosis* (Mtb), the pathogen of human tuberculosis (TB), is a global health burden with high morbidity and mortality rates [1]. Despite the development of new antimicrobial strategies for TB, little progress has been made in improving or replacing short-term chemotherapy (directly observed treatment, short course), which comprises isoniazid (INH), rifampicin, pyrazinamide, and ethambutol during the initial 2 months, followed by INH and rifampicin for 4 months, as the first-line regimen for drug-sensitive TB [2, 3]. The treatment of drug-resistant cases is difficult and can require > 2 years of drugs with severe side effects. The increasing incidences of multidrug-resistant (MDR) and extremely drug-resistant TB are worrisome [2, 4, 5]. Moreover, nontuberculous mycobacteria (NTM) include > 170 species and are major pathogens of emerging respiratory infections in immunocompromised and immunocompetent subjects [6, 7]. The prevalence and incidence of NTM pulmonary infections are increasing worldwide and often intractable to treat [8–10]. NTM treatment with standard antimicrobial regimens is challenging, takes longer than TB treatment, and has a low cure rate because of antibiotic resistance and toxicity [11–13]. There is an urgent need for innovative host-directed therapeutics (HDT) based on deciphering an in-depth molecular mechanisms underlying host–pathogen interactions that could be geared for efficient protective responses against TB and NTM infections.

Immune-metabolic adaptations are rewired in macrophages and immune cells during mycobacterial infections. The remodeling of immunometabolism can shape the host defensive responses to intracellular mycobacteria, thus affecting the outcomes of infections [14–16]. Early studies highlighted the function of the immunometabolite itaconic acid (also known as methylenesuccinic acid) in decarboxylating *cis*-aconitate, which exerts an antimicrobial effect on *Salmonella enterica* and Mtb by covalent inhibition of bacterial isocitrate lyase [17, 18]. Immune-responsive gene 1 (IRG1), a mitochondrial enzyme that catalyzes the production of itaconate in myeloid cells, controls Mtb infection by preventing excessive neutrophil-driven immunopathology [19]. In addition, autocrine/paracrine signaling by tumor necrosis factor (TNF)-α and interleukin (IL)-6, which are generated by bystander cells, leads to the juxtaposition of bacterial phagosomes with mitochondria, thereby activating IRG1 signaling and an itaconate-mediated antimicrobial effect against *M. avium* (Mav) infection [20]. Furthermore, increasing efforts have been made to develop more cell-permeable derivatives of itaconate than the natural itaconate with polar structure, while they retain the immunoregulatory function [21–23]. Indeed, dimethyl itaconate (DMI) and 4-octyl itaconate (OI) ameliorate excessive inflammation and pathologic responses in models of autoimmune and inflammatory disorders including sepsis, mastitis, neuroinflammation, and psoriasis [22–26]. Mounting evidence showed that DMI is able to induce a robust electrophilic stress response, and activates NF-E2 p45-related factor 2 (Nrf2) and the expression of genes encoding its downstream biomolecules including *Nqo1* and *Hmox1* in the lipopolysaccharide (LPS)-stimulated murine macrophages [24, 25]. In addition, DMI showed a strong inhibitory effect upon the production of IL-6, IL-10, and interferon (IFN)-β, although these effects are independent of Nrf2 [24, 25]. DMI-mediated anti-inflammatory response is also mediated through a well-known negative regulator of toll-like receptor signaling, activating transcription factor 3/IκBζ pathway [24]. Interestingly, DMI is not directly metabolized into itaconate, but it potentiates an increase in the itaconate level in LPS-stimulated macrophages [26]. Despite this, it remains largely undefined whether and how DMI modulates antimicrobial host defense against Mtb, NTM, and drug-resistant mycobacterial infections.

In this study, we aimed to evaluate whether DMI exerts host defensive functions and how it achieved protective immune reactions during mycobacterial infections. We examined whether DMI increases antimicrobial host responses against Mtb, *M. bovis* Bacillus Calmette–Guérin (BCG), Mav, and multidrug-resistant (MDR)-Mtb infections. Although DMI per se did not exhibit direct antimicrobial effects against Mtb, BCG, or Mav, it showed potent antimicrobial activities in macrophages and in vivo. Mechanistically, DMI played multiple roles in the activation of innate immune defenses, i.e., maintenance of inflammatory homeostasis, signal transducer and activator of transcription 3 (STAT3) signaling, and activation of autophagy. These data offer DMI as an effective therapeutic candidate of HDT against Mtb and NTM infections by modulating multifaceted innate immune pathways.

## Materials and methods

### Mycobacterial strains and cultivation

Mtb H37Rv was supplied by R.L. Friedmann (University of Arizona, Tucson, AZ). BCG, MDR-Mtb (KMRC-00116-00150), and Mav (ATCC 25291) were acquired from the Korean Mycobacterium Resource Center in the Korean Institute of Tuberculosis (Osong, South Korea). Mycobacteria were cultured in Middlebrook 7H9 (Difco, 271310) medium supplemented with 10% oleic albumin dextrose catalase (OADC; BD Biosciences, San Diego, CA, 212240), 0.5% glycerol, and 0.05% Tween-80 (7H9-OADC) on a rotary shaking incubator (140 rpm) at 37°C to an OD_600_ of 0.4–0.6. Mtb expressing red fluorescent protein (Mtb-ERFP) was cultivated in 7H9-OADC supplemented with 50 µg/ml kanamycin (Sigma-Aldrich, St. Louis, MO, 60615). Bacterial cultures were harvested, and the pellets were washed with phosphate-buffered saline (PBS; LPS solution, Daejeon, Korea, CBP007B) by sequential centrifugation at 2090×*g* (3000 rpm) for 30 min. To separate bacteria into single cells, pellets resuspended in PBS with 0.1% Tween-80 were subjected to repeated rounds of sonication. The resulting bacterial suspensions were aliquoted and stored at −80°C. Colony-forming units (CFUs) were counted on Middlebrook 7H10 agar (Difco, 262710).

### Mice

Wild-type (WT) C57BL/6 mice (Samtako Bio, Gyeonggi-do, South Korea) were obtained at 6–8 weeks of age and maintained under a 12 h:12 h light:dark cycle and specific-pathogen-free conditions. Information on *Atg7*-floxed mice and *Atg7*-lacking mice is available elsewhere [27]. The mice were 6–8 weeks old at the time of the experiments and were matched by sex. The animal experiments and handling were conducted following the ethical guidelines of Chungnam National University School of Medicine and were approved by the Institutional Animal Care and Use Committee (202109A-CNU-180; Daejeon, South Korea) and the South Korean Food and Drug Administration.

### Isolation of bone marrow-derived macrophages (BMDMs) and peritoneal macrophages (PMs)

BMDMs were collected from the femur and tibia of 6–8-week-old mice and cultured for 4–5 days in Dulbecco’s modified Eagle’s medium (DMEM; Lonza, Walkersville, USA, BE12-60fF) supplemented with 10% fetal bovine serum (Gibco, Grand Island, NY, 16000-044) and penicillin streptomycin amphotericin mixture (Lonza, 17-745E) containing 25 ng/ml macrophage colony-stimulating factor (R&D Systems, Minneapolis, MN) at 37°C in 5% CO_2_. For the isolation of PMs from *Atg7*-floxed and *Atg7*-lacking mice (8-week-old), intraperitoneal injection of mice were performed using 1 ml of 3% Brewer thioglycollate (BD Biosciences, 211716). After 3 days from injection, the isolation of cells were conducted by flushing out the peritoneal cavity with 10 ml of Dulbecco's PBS (DPBS; Cytiva Hyclone^TM^, Marlborough, MA, SH30028.02) containing 10% fetal bovine serum. The appropriate number of cells were seeded and incubated for 1 day in DMEM supplemented with 10% fetal bovine serum and penicillin streptomycin amphotericin mixture.

### Experimental infection

Bacterial cells stored at −80°C were thawed and diluted in DPBS containing 0.05% Tween-80. Vial containing bacterial cells was sonicated to a bath sonicator 3 times for 30 s. Infection of BMDMs and PMs at the indicated multiplicities of infection (MOI) of Mtb, BCG, or Mav was conducted for 4 h. The remaining bacteria around cells were removed by washing with DPBS, and infected cells were incubated in fresh DMEM for the indicated times. For in vivo  infection, mice were anesthetized and intranasally infected with mycobacteria (Mtb: 5 × 10^4^ CFU/mouse; BCG: 1 × 10^7^ CFU/mouse; Mav: 1 × 10^7^ CFU/mouse; MDR-Mtb: 5 × 10^3^ CFU/mouse). To estimate the bacterial burden, mice were euthanized at 7 days post infection (dpi), and lungs were harvested, homogenized in DPBS, serially diluted, and spotted on 7H10 agar. After incubation for 2–3 weeks, colonies were counted.

### Reagents and antibodies

DMI (592498), bafilomycin A1 (Baf-A1; B1793), d-(+)-glucose (glucose; G7021), sodium acetate (S8750), and β-cyclodextrin (H5784) were purchased from Sigma-Aldrich (St. Louis, MO). For Western blotting, anti-pSTAT3 (9145S), anti-STAT3 (9139S), anti-microtubule-associated protein 1 light chain 3 β (LC3) (L7543), anti-ACTIN (5125S), anti-mouse IgG (7076S), and anti-rabbit IgG (7074S) antibodies were purchased from Cell Signaling Technology (Danvers, MA). For immunofluorescence analysis, an anti-LC3 (PM036) antibody was obtained from Medical & Biological Laboratories International, and an anti-lysosomal-associated member protein 1 (LAMP1; SC-19992) antibody was obtained from Santa Cruz Biotechnology. Alexa Fluor 488-conjugated anti-rabbit IgG (A11034) and Alexa Fluor 594-conjugated anti-rat IgG (A21209) antibodies were from Invitrogen (Waltham, MA). Fluoromount-G with 4′-6-diamidino-2-phenylindole (DAPI) (00-4959-52) was obtained from Invitrogen (Waltham, MA).

### CFU assay

To analyze bacterial survival in murine macrophages, Mtb-, BCG-, or Mav-infected cells (MOI 1) were incubated for 4 h and washed with DPBS to discard extracellular bacteria. The infected cells were incubated in fresh medium for the indicated periods. Thereafter, the cells were lysed in sterile distilled water for 40 min, and intracellular bacteria were collected. Cell lysates were diluted with DPBS and spotted on Middlebrook 7H10 agar containing 10% OADC. Colonies were counted to assess intracellular bacterial viability after 2–3 weeks.

### Histology and immunohistochemistry

Lungs were removed from Mtb-infected mice, fixed in 10% formalin, and embedded in paraffin wax. Paraffin blocks were sectioned (4 µm) and stained with hematoxylin and eosin (H&E) as described previously [28]. The inflamed area was quantified by total field scanning of lung tissues, and the mean fluorescence intensity of the red threshold was examined using FIJI software.

### Determination of dose–response curves

The half-maximal inhibitory concentration (IC_50_) values were determined following the CLSI guidelines [29]. Mtb, BCG, and Mav were cultured in 7H9-OADC to an OD_600_ of 0.4–0.6 followed by harvesting, washing twice with PBS supplemented with 0.02% Tween-80, and vortexing 3 times for 5 s with 30–50 glass beads of diameter 2 mm. Finally, low-speed centrifugation (250×*g*) leaves only single bacterial cells in supernatant. Inoculum was prepared from this supernatant. All wells contained 7H9-OADC, 7H9 medium supplemented with 0.5% glycerol, 0.02% Tween 80 and 10 mM glucose (7H9-glucose), or 10 mM sodium acetate (7H9-acetate). Then all wells were added by each inoculum of Mtb, BCG, or Mav within each media with an OD_600_ of 0.005/ml except for the negative control (medium only). DMI, β-cyclodextrin (SC), and INH were two-fold serially diluted 20 times (DMI, β-cyclodextrin) or 10 times (INH) in flat-bottom clear 96-well plates, ranging from 50 mM to 95 nM (DMI) or from 10 μM to 19.5 nM (INH) containing Mtb, BCG, or Mav in a final volume of 100 µl, and then incubated for 5 to 7 days (7H9-OADC and -glucose) or 10 to 14 days (7H9-acetate) at 37°C. OD_600_ values were determined using the VersaMax microplate reader (Molecular devices, Sunnyvale, CA). IC_50_ values were calculated from the OD_600_ values using Prism 8.0 software (GraphPad Inc., La Jolla, CA).

### RNA preparation and quantitative real-time PCR (qRT-PCR)

Total RNA was isolated from BMDMs, PMs, or lung tissue homogenates using TRIzol reagent (Invitrogen, Waltham, MA, 15596026) in accordance with the manufacturer’s instructions. cDNA was prepared from total RNA using Reverse Transcription Master Premix (ELPIS Biotech, Daejeon, South Korea, EBT-1515) following the manufacturer’s protocols. qRT-PCR was conducted on the Rotor-Gene A 2plex System (Qiagen, Hilden, Germany, 9001620) using cDNA, primer pairs specific to the genes of interest, and SYBR Green Master Mix (Qiagen, 218073) according to the manufacturer’s instructions. Relative mRNA levels were analyzed by the 2^−ΔΔ^ threshold cycle method and normalized to those of *Gapdh*. The primer sequences used are as follows: *Il1b* forward: 5′-TGACGGACCCCAAAAGATGA-3′, reverse: 5′-AAAGACACAGGTAGCTGCCA-3′; *Il6* forward: 5′-ACAAAGCCAGAGTCCTTCAGA-3′, reverse: 5′-TGGTCCTTAGCCACTCCTTC-3′; *Il10* forward: 5′-GCTCTTGCACTACCAAAGCC-3′, reverse: 5′-CTGCTGATCCTCATGCCAGT-3′; *Tnf* forward: 5′-CCCACGTCGTAGCAAACCAC-3′, reverse: 5′-GCAGCCTTGTCCCTTGAAGA-3′; *Ifng* forward: 5′-CGGCACAGTCATTGAAAGCC-3′, reverse: 5′-TGCATCCTTTTTCGCCTTGC-3′; *Csf2* forward: 5-CTG GCC CCA TGT ATA GCT GA-3′, reverse: 5′-TCC TCC TCA GGA CCT TAG CC-3′; *Gapdh* forward: 5′-TGGCAAAGTGGAGATTGTTGCC-3′, reverse: 5′-AAGATGGTGATGGGCTTCCCG-3′.

### Enzyme-linked immunosorbent assay (ELISA)

The supernatant of mouse BMDMs or lung lysates was stored at −80°C. The levels of the proinflammatory cytokines IL-6 (555240) and IL-10 (555252) were estimated using the Mouse BD OptEIA Set ELISA Kit (BD Biosciences) according to the manufacturer’s instructions.

### Immunofluorescence analysis

BMDMs were grown on coverslips in 24-well plates followed by infection with Mtb-ERFP (MOI 5) for 4 h. The solvent control (SC), DMI (100 μM) or Baf-A1 (100 nM) was then treated within the freshly changed media for the indicated times. After treatment, the upper medium was discarded, and cells on coverslips were washed three times in DPBS followed by fixation in 4% paraformaldehyde for 10 min. Thereafter, the cells were permeabilized in 0.25% Triton X-100 for 10 min and incubated overnight at 4℃ with anti-LC3 (1:400 diluted) and anti-LAMP1 (1:400 diluted) primary antibodies. The cells were washed three times with DPBS and reacted for 2 h with the secondary antibody at room temperature. Fluoromount-G with DAPI was used to stain nuclei and mount the cells. For imaging acidic states of phagolysosome, BMDMs infected with Mtb-ERFP (MOI 5) were treated with SC or DMI (100 μM) for the indicated times, followed by the incubation of cells with LysoView 633 (Biotium, Fremont, USA; 70058) for 30 min at 37°C. Cells were DPBS-washed two times and mounted by fluoromount-G with DAPI. The excitation of LysoView 633 was performed by the 633 nm laser, and pictured at 645–750 nm. To determine the autophagic flux in BMDMs, cells were transduced with retroviruses expressing a tandem-tagged mCherry-enhanced green fluorescent protein (EGFP)-LC3B for 24 h and then treated with DMI in the presence or absence of Baf-A1 for 24 h. Cells with tandem LC3B plasmid was detected by confocal microscopy.

### Transmission electron microscopy (TEM)

Cells were scraped from plates and collected by centrifugation. Samples were sequentially fixed in 3% glutaraldehyde and 1% osmium tetroxide, cooled on ice for 1 h, washed with 0.1 M cacodylate buffer (pH 7.2) containing 0.1% CaCl_2_, and dehydrated in an ethanol and propylene oxide series. Next, samples were embedded in Epon 812 mixture and polymerized at 60°C for 36 h. Using the ULTRACUT UC7 ultramicrotome (Leica Biosystems, Vienna, Austria), sections of 70 nm thickness were cut and mounted on 75-mesh copper grids. Sections were counterstained with uranyl acetate and lead citrate for 10 min and 7 min, respectively, and examined using the KBSI Bio-High Voltage EM (JEM-1400 Plus at 120 kV and JEM-1000BEF at 1000 kV; JEOL Ltd., Tokyo, Japan).

### Western blotting

BMDM lysates were collected in Protein 5× Sample Buffer (ELPIS BIOTECH, EBA-1052) diluted with RIPA buffer (150 mM sodium chloride, 1% Triton X-100, 0.1% SDS, 1% sodium deoxycholate, 50 mM Tris-Cl at pH 7.5, and 2 mM EDTA) supplemented with protease (04693132001) and phosphatase (11836170001) inhibitor cocktails (Roche, Mannheim, Germany). Samples were boiled for 10 min on a heating block and cooled on ice for 10 min. The samples were resolved by SDS-PAGE and transferred to a nitrocellulose (Pall Corporation, NY, 66485) or PVDF (Millipore, Burlington, MA, IPVH0001) membrane at 200 mA for 2 h. To prevent nonspecific binding, membranes were incubated in blocking solution with 1% BSA in TBST for 30 min at room temperature and reacted overnight with anti-pSTAT3, -STAT3, or -ACTIN primary antibody at 4°C. The membranes were reacted with the appropriate horseradish peroxidase-conjugated secondary antibodies for 1 h at room temperature. Immunoreactive bands were visualized with ECL reagent from the Chemiluminescence Assay Kit (Millipore, WBKL S0500), and the appropriate bands were detected using the UVitec Alliance mini-chemiluminescence device (UVitec, Rugby, UK). Band intensities were measured using Image J software and normalized to that of ACTIN.

### Statistical analysis

Statistical analysis was conducted using Prism 8.0 for Windows (GraphPad Software Inc., San Diego, CA). The unpaired Student’s *t*-test or Mann–Whitney U test was used to compare two groups and one-way ANOVA for three or more groups. Data are means ± standard deviation (SD) or ± standard error of the mean (SEM). Statistical significance is indicated as *p < 0.05, **p < 0.01, and ***p < 0.001.

## Results

### DMI induces antimicrobial activity against Mtb, BCG, and Mav in vitro and in vivo

**Fig. 1:**
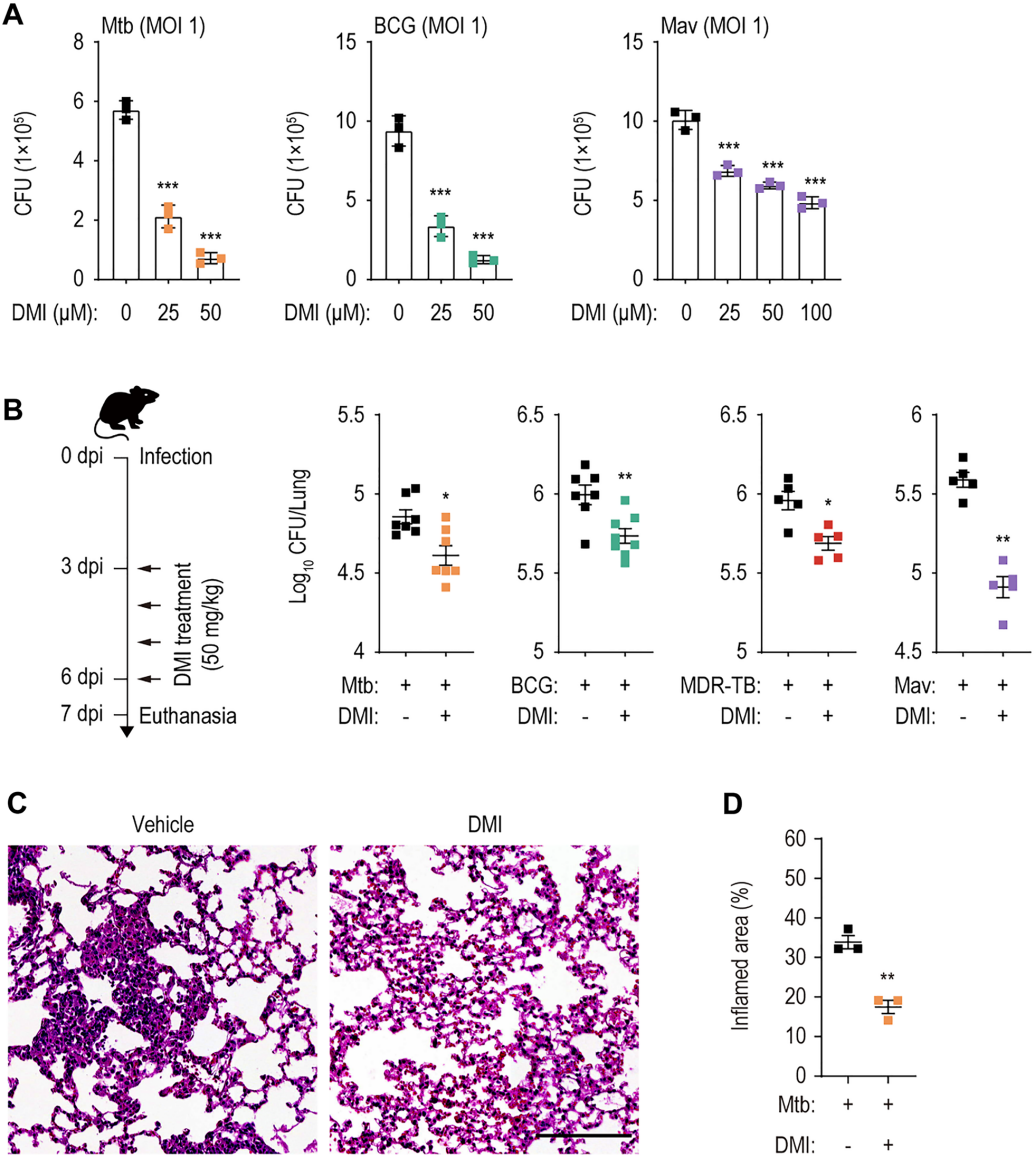
DMI-treatment inhibits the both in vitro and in vivo mycobacterial survival. **A** Intracellular survival of mycobacteria in BMDMs infected with Mtb, BCG, or Mav (MOI 1). BMDMs were infected with Mtb (left panel), BCG (mid panel), or Mav (right panel). After 4 h, cells were washed with pre-warmed DPBS and treated with SC or indicated concentration of DMI. At 3 dpi, cells were lysed and used to a CFU assay to examine the intracellular survival of Mtb, BCG, or Mav. **B** Mice were intranasally infected with Mtb (5 × 10^4^ CFU, n = 7–8 per group), BCG (1 × 10^7^ CFU, n = 7–8 per group), MDR-Mtb (5 × 10^3^ CFU, n = 5 per group), or Mav (1 × 10^7^ CFU, n = 5 per group), followed by treatment with vehicle or DMI (50 mg/kg) by intraperitoneal (i.p.) injection, and euthanized as depicted schematic diagram of experimental schedule (left panel). The dissected lungs from mice were subjected to analyze the bacterial burden by CFU assay. **C**, **D** Mice (n = 3 per group) were infected with Mtb (5 × 10^4^ CFU) followed by treatment with vehicle or DMI (50 mg/kg) by i.p. injection. At 28 dpi, lungs were harvested to determine the inflamed area. Representative histopathological images (**C**, scale bar = 300 μm) and quantitative analysis for the inflamed area of the lung tissues from mice using H&E (**D**). Statistical analysis was determined with one-way ANOVA test with Tukey’s multiple comparisons (**A**) and Mann–Whitney U test (**B**, **D**). Data are representative of at least three independent experiments, and error bars denote ± SD (**A**) or ± SEM (**B**, **D**). *CFU* colony forming unit, *DMI* dimethyl itaconate, *MOI* multiplicities of infection, *dpi* days post infection, *MDR-TB* MDR-Mtb. *p < 0.05, **p < 0.01, and ***p < 0.001

IRG1, a mitochondrial enzyme that produces itaconate via decarboxylation of *cis*-aconitate [17], protects against Mtb infection in a mouse model by controlling excessive pathological inflammatory responses and neutrophil recruitment [19]. To assess the antimicrobial effect of DMI on Mtb, BCG, and NTM infections, we infected murine BMDMs with Mtb H37Rv, BCG, or Mav at an MOI of 1 and monitored bacterial survival at 72 h post-infection. DMI treatment caused dose-dependent suppression of intracellular Mtb, BCG, and Mav (Fig. 1A). To examine whether DMI directly kills mycobacteria, we first determined the IC_50_ values and generated dose–response curves in culture conditions of standard medium 7H9-OADC. The IC_50_ values of DMI for Mtb, BCG, and Mav were 866 µM, 1.2 mM, and 3.8 mM, respectively, whereas the IC_50_ of INH was 154 nM. Compared with INH, the IC_50_ values of DMI were over 5000-fold for all tested mycobacterial strains (Additional file 1: Fig. S1). Isocitrate lyase, a target of itaconate, is an essential component of glyoxylate shunt found in many pathogens including mycobacteria [30–32]. This metabolic pathway is activated under carbon-limiting conditions through a bypass of TCA cycle [33, 34]. We thus determined whether DMI induces direct antibacterial activities against Mtb, BCG, and Mav, depending on culture conditions containing different carbon sources, i.e., glucose or acetate. When IC_50_ values were compared between glucose- and acetate-containing culture conditions, DMI showed significantly greater IC_50_ values for Mtb (7H9-glucose, 2.9 mM; 7H9-acetate, 3.3 mM), for BCG (7H9-glucose, 3 mM; 7H9-acetate, 1.5 mM), and for Mav (7H9-glucose, 5 mM; 7H9-acetate, 4.1 mM), when compared to those of INH for Mtb (7H9-glucose, 183 nM; 7H9-acetate, 190 nM) (Additional file 1: Fig. S2). Therefore, the intracellular antimicrobial responses to DMI may not be caused by its direct inhibition of mycobacterial growth.

C57BL/6 mice were infected intranasally with Mtb (5 × 10^4^ CFU/mouse), BCG (1 × 10^7^ CFU/mouse), MDR-Mtb (5 × 10^3^ CFU/mouse), or Mav (1 × 10^7^ CFU/mouse) and treated intraperitoneally with 50 mg/kg DMI or the vehicle control for 4 days (Fig. 1B, left). We found that DMI treatment significantly enhanced in vivo antimicrobial effects during infection with Mtb, BCG, MDR-Mtb, or Mav in mouse lung tissues (Fig. 1B). Pronounced differences were observed in the in vivo bacterial loads of all four different strains in the lung tissues from the infected mice between DMI-treated and vehicle-treated groups (Fig. 1B). In addition, DMI significantly inhibited granulomatous lesions in the lung tissues of Mtb-infected mice (Fig. 1C, D). Taken together, these data strongly suggest that DMI significantly suppresses the pulmonary bacterial loads and inflammatory lesions in mice during mycobacterial infections.

### DMI modulates inflammatory and protective cytokine generation in macrophages and in the lung tissues from infected mice

**Fig. 2:**
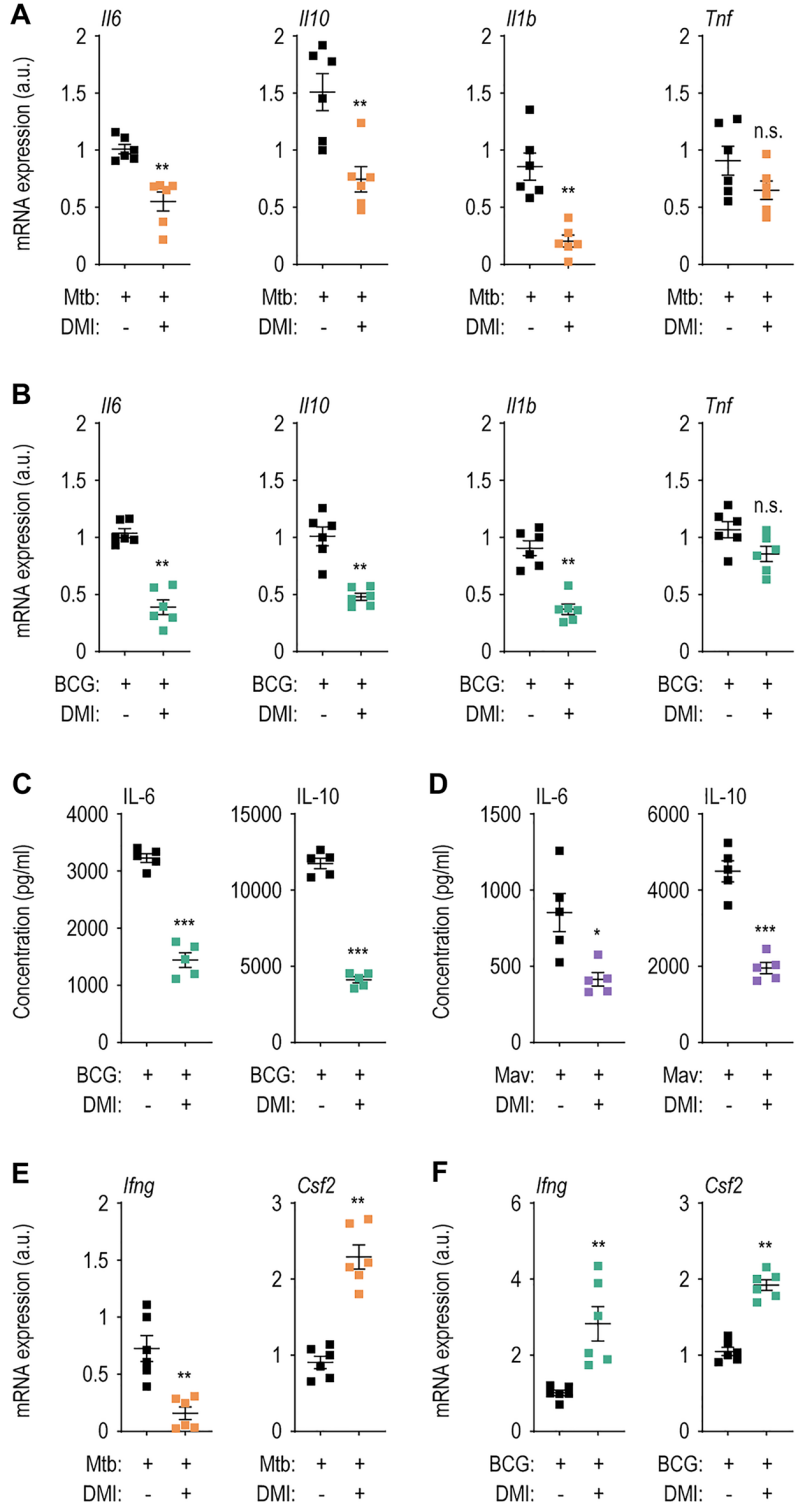
The treatment with DMI suppresses the expression of inflammatory cytokines and increases the level of protective cytokines in lung tissues from mycobacteria-infected mice. WT mice (n = 5–6 per group) were infected intranasally with Mtb (5 × 10^4^ CFU), BCG (1 × 10^7^ CFU), or Mav (1 × 10^7^ CFU) followed by treatment with vehicle or DMI (50 mg/kg) in accordance with experimental schedule, and monitored at 7 dpi. Lung tissues from Mtb- (**A**) or BCG- (**B**) infected mice were used to qRT-PCR analysis to estimate the mRNA expression of *Il6*, *Il10*, *Il1b*, and *Tnf*. **C**, **D** The supernatants from lung lysates separated from BCG- (**C**) or Mav- (**D**) infected mice were used to ELISA analysis. Lung tissues from Mtb- (**E**) or BCG- (**F**) infected mice were used to qRT-PCR anlaysis to examine the mRNA expression of *Ifng* and *Csf2*. Mann–Whitney U test was used to examine the statistical analysis and the results were shown as means ± SEM from at least three independent experiments performed. *DMI* dimethyl itaconate, *n.s.* not significant, *a.u.* arbitrary unit. *p < 0.05, **p < 0.01, and ***p < 0.001

Because DMI reduced the bacterial burden and lung pathological lesions at the early (7 dpi) stages of infection (Fig. 1), we examined its effect on lung inflammatory responses during mycobacterial infection. We thus compared the expression levels of proinflammatory cytokines *Tnf*, *Il6*, *Il1b*, and the anti-inflammatory cytokine *Il10*, in the lung tissues between the DMI-treated and vehicle-treated control mice during infection. The *Il6*, *Il10*, and *Il1b* levels were significantly suppressed, whereas *Tnf* mRNA level was not reduced, by DMI treatment in the lung tissues from mice infected with Mtb and BCG (Fig. 2A, B; at 7 dpi). Consistent with the mRNA data, the IL-6 and IL-10 protein levels were markedly reduced in the supernatants of lung lysates from DMI-treated compared with control mice infected with BCG and Mav (Fig. 2C, D). Thus, both IL-6 and IL-10 levels were specifically modulated by DMI treatment in vivo during mycobacterial infection. We next assessed whether DMI modulates the mRNA levels of IFN-γ and granulocyte–macrophage colony-stimulating factor (GM-CSF), which are known as protective cytokines associated with anti-mycobacterial host defense [35–38] in the infected lungs from mice during Mtb and BCG infections. As shown in Fig. 2E, F, the *Csf2* levels were significantly upregulated by DMI treatment in both Mtb- and BCG-infected lung tissues. However, the mRNA level of *Ifng* was significantly reduced in Mtb-infected lungs, whereas it was increased in the lung tissues from BCG-infected mice (Fig. 2E, F). These data suggest that DMI treatment differentially modulated protective cytokine generation in the lung tissues depending on the mycobacterial strains.

**Fig. 3:**
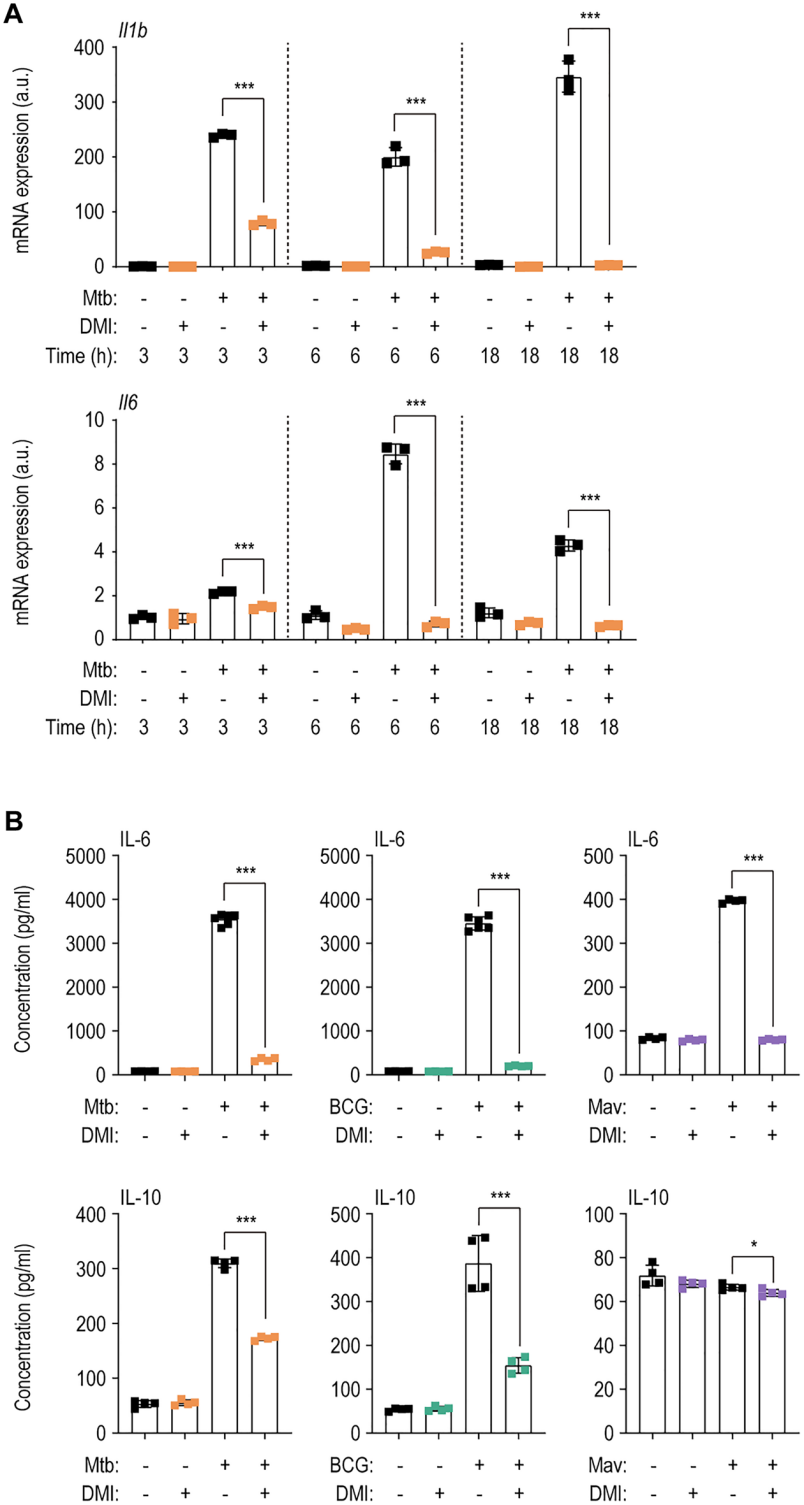
DMI-treatment reduces the expression level of inflammatory cytokines during various mycobacterial infections. **A** Mtb (MOI 3)-infected BMDMs were incubated in the freshly changed media treated with SC or 100 μM of DMI. Cells were lysed at the indicated time points (3, 6, or 18 ﻿h) and used to qRT-PCR analysis to estimate the expression level of *Il1b* and *Il6*. **B** The supernatants from the BMDMs infected with Mtb (left panel), BCG (mid panel), or Mav (right panel) were harvested at 18 h post-infection and used to ELISA to examine the cytokine level of IL-6 and IL-10. Statistical analysis was conducted with one-way ANOVA test with Tukey’s multiple comparisons (**A**) and unpaired Student’s *t*-test (**B**). Data shown as means ± SD from two independent experiments conducted in triplicate. DMI, dimethyl itaconate. *p < 0.05 and ***p < 0.001

We further examined the effect of DMI on Mtb-induced mRNA levels of inflammatory cytokines (*Tnf*, *Il1b*, and *Il6*) in macrophages (Fig. 3A and Additional file 1: Fig. S3A). Mtb-induced *Tnf* expression was significantly increased by DMI at 6 h, but decreased at 18 h, in BMDMs (Additional file 1: Fig. S3A). However, Mtb-induced *Il1b* and *Il16* levels were markedly suppressed by DMI at 3, 6, and 18 h (Fig. 3A). Additionally, the Mav-induced *Il6* and *Il1b* levels were markedly decreased at 3–18 h, as with Mtb infection (Additional file 1: Fig. S3B). Moreover, DMI markedly suppressed IL-6 and IL-10 production in BMDMs infected with Mtb, BCG, or Mav, compared with those induced by the infection alone (Fig. 3B). Therefore, DMI treatment regulates inflammatory homeostasis in vivo and in macrophages during mycobacterial infection.

### DMI promotes the activation of autophagy and autophagic flux in BMDMs

**Fig. 4:**
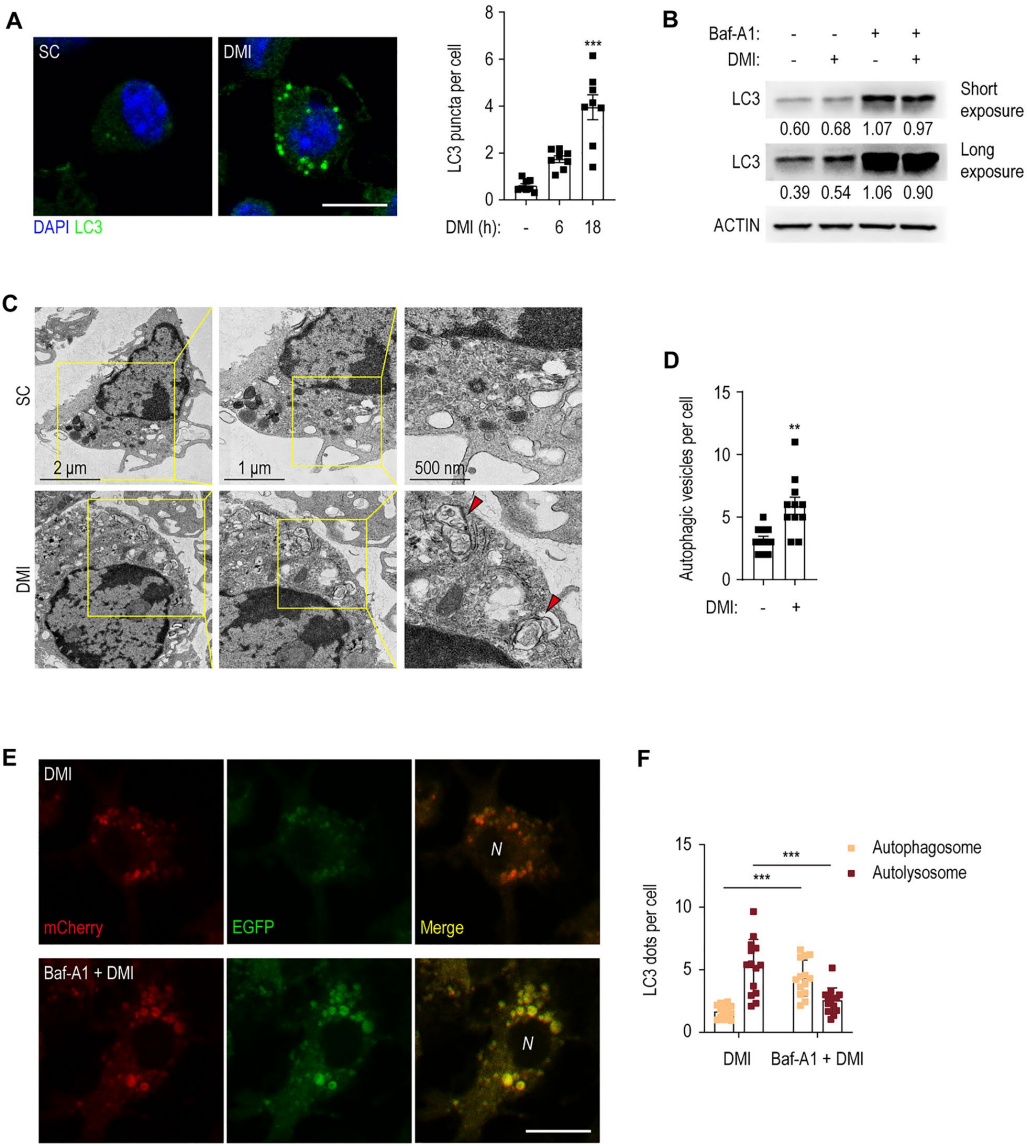
DMI-treatment increases the activation of autophagy and autophagic flux. **A** BMDMs were treated with SC or DMI (100 μM) for the indicated times and stained with anti-LC3 (green) and DAPI (for nuclei; blue). Representative immunofluorescence microscopy images (left panel) and quantitation of LC3 puncta per cell (right panel). At least 100 cells in independent 8 fields were counted in each group from two different experiments. Scale bar, 2 μm. **B** BMDMs were pre-incubated with or without Baf-A1 (100 nM) for 2 h and followed by treatment with SC or DMI (100 μM) for 8 h. LC3 and ACTIN levels were evaluated by Western blot analysis. **C**, **D** BMDMs were treated with SC or DMI (100 μM) for 18 h. Representative TEM image (**C**) and quantitation of autophagic vesicles per cell (**D**). **E**, **F** BMDMs were transduced with retroviruses expressing a tandem-tagged mCherry-EGFP-LC3B. After 24 h, cells were pre-incubated with or without Baf-A1 (100 nM) for 2 h and followed by treatment with SC or DMI (100 μM) for 24 h. Cells were collected and mCherry or EGFP expressing LC3B were detected by confocal microscopy. Representative immunofluorescence microscopy images (**E**) and quantitation of LC3 dots per cell (**F**). Scale bars, 2 μm. Statistical analysis was determined with one-way ANOVA test with Tukey’s multiple comparisons (**A**), unpaired Student’s *t*-test (**D**), and two-way ANOVA test with Sidak’s multiple comparisons (**F**). Data are representative of at least three independent experiments, and error bars denote ± SD. *SC* solvent control, *DMI* dimethyl itaconate, *Baf-A1* bafilomycin A1, *N* nucleus. **p < 0.01 and ***p < 0.001

DMI induces cellular autophagy to inhibit NLRP3-mediated pyroptosis [39]. We thus examined whether DMI activates autophagy to enhance antibacterial responses during infection. DMI robustly increased LC3 punctate structures in BMDMs in a time-dependent manner (Fig. 4A). In addition, Western blotting revealed that DMI caused slight but significant conversion of cytosolic LC3-I to autophagosome-associated LC3-II (Fig. 4B). Moreover, pre-treatment of BMDMs with Baf-A1 significantly increased the level of lipidated LC3-II, indicating that DMI increases autophagic flux (Fig. 4B, lane 4).

Ultrastructural analysis by TEM showed that DMI significantly increased the number of autophagic vesicles (autophagosomes and autolysosomes) in BMDMs (Fig. 4C, D). We further transduced BMDMs with a retroviral vector containing a mCherry-EGFP-LC3B prior to DMI treatment. The number of red punctate structures (mCherry; acid stable) denoting the state of autolysosomes (acidic pH quenches GFP fluorescence) were significantly increased. However, pre-treatment with Baf-A1, an inhibitor of the lysosomal V-ATPase, prevented this effect in DMI-treated BMDMs (Fig. 4E, F). These data confirm that DMI promotes the activation of autophagy and autophagic flux in BMDMs.

### DMI-mediated autophagy enhances phagosomal maturation of Mtb

**Fig. 5:**
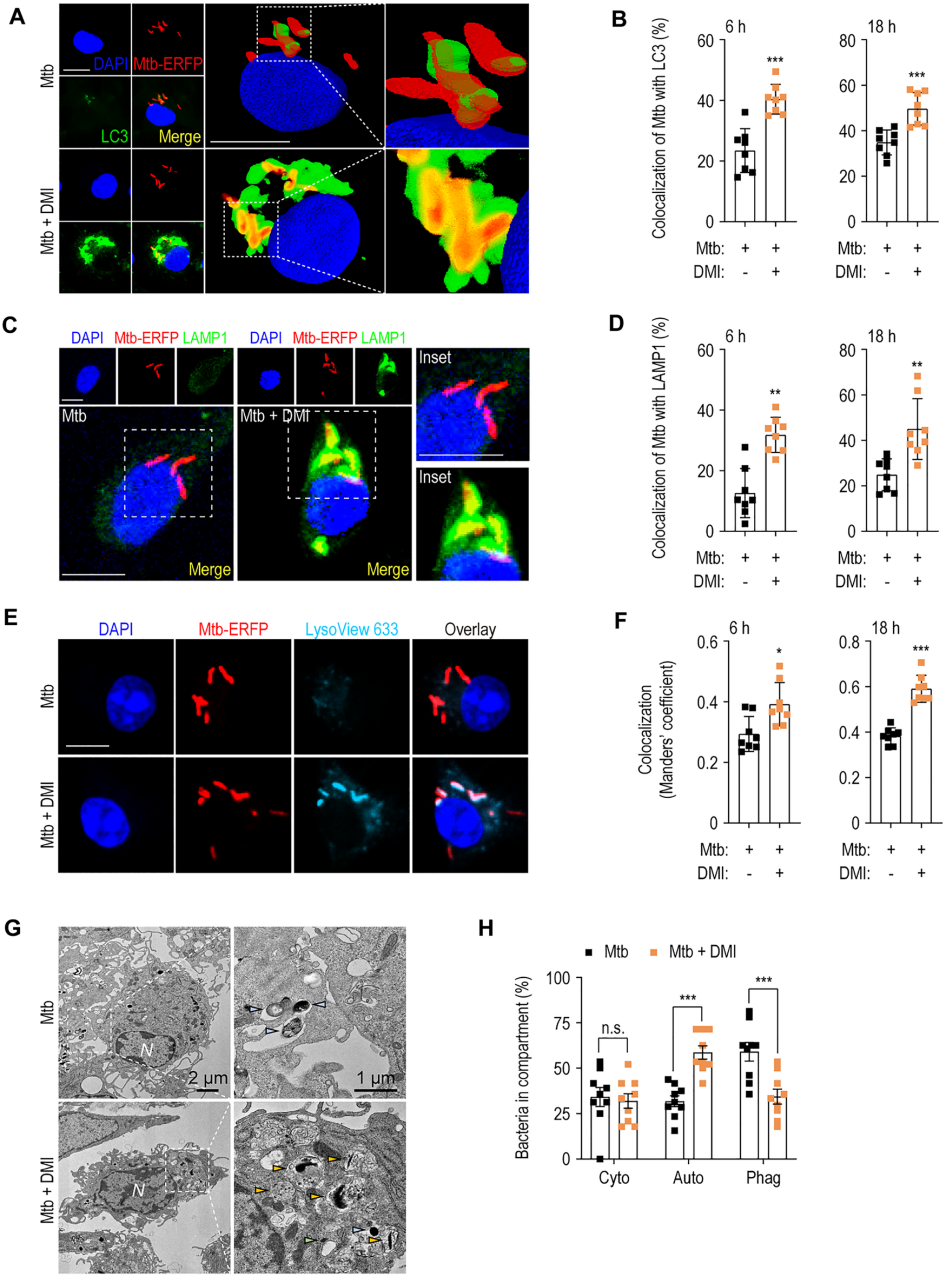
DMI enhances antibacterial autophagy against infection with Mtb in BMDMs. **A**–**D** BMDMs were infected with Mtb-ERFP (MOI 5) and followed by treatment with SC or DMI (100 μM) for the indicated times. Mtb-ERFP (red), Alexa Fluor 488-conjugated LC3 (green, **A**) or LAMP1 (green, **C**), DAPI (for nuclei, blue) were detected by confocal microscopy. Representative immunofluorescence images (**A** for LC3, **C** for LAMP1) and quantitation of colocalization of Mtb-ERFP with LC3 (**B**) or LAMP1 (**D**) were shown. Scale bars, 2 μm. **E**, **F** Mtb-ERFP-infected (MOI 5) BMDMs were treated with SC or DMI (100 μM) for the indicated times. Mtb-ERFP (red), Lysoview 633 (skyblue), and DAPI (for nuclei, blue) were detected by confocal microscopy. Representative immunofluorescence images (**E**) and quantitation of colocalization of Mtb-ERFP with LysoView 633 (**F**) using Manders’ coefficient were assessed. **G**, **H** BMDMs were infected with Mtb (MOI  5) and followed by treatment with SC or DMI (100 μM) for 18 h. Representative TEM images (**G**) and quantitation of bacteria in compartment (**H**). Bacteria in cytosol (light green), autophagosomes (orange), and phagosomes (pale blue) were marked as indicated. Unpaired Student’s *t*-test was used to examine the statistical analysis and the results were shown as means ± SD from at least three independent experiments performed. *N* nucleus, *DMI* dimethyl itaconate, *n.s.* not significant, *Cyto* cytosol, *Auto* autophagosomal/autolysosomal structure, *Phag* phagosome. *p < 0.05, **p < 0.01, and ***p < 0.001

We next examined the effect of DMI on phagosomal maturation of Mtb in BMDMs. To examine this, we performed immunostaining and assessed the bacteria colocalization with LC3-positive autophagosomes and lysosomal structures in the Mtb-ERFP-infected BMDMs. DMI treatment in Mtb-ERFP-infected BMDMs significantly increased the colocalization of endogenous LC3-positive autophagosomal structures and Mtb-ERFP at 6 (early) and 18 (late) h (Fig. 5A, B). Additionally, DMI significantly increased the colocalization of Mtb-ERFP with LAMP1-positive lysosomal vesicles in BMDMs at the same time points (Fig. 5C, D). Moreover, we assessed whether DMI enhanced the Mtb captured in acidic compartment by staining with LysoView 633 dye, which is a highly sensitive pH sensor of the acidified lysosomes [40, 41]. As shown in Fig. 5E, F, we found that DMI treatment results in the significantly increased colocalization of Mtb-ERFP and LysoView 633^+^ acidic compartment.

We next examined the changes of ultrastructural findings by DMI treatment in Mtb-infected macrophages. TEM analysis showed that bacteria-containing autophagosomal–autolysosomal structures, where bacteria are localized within the host vesicles surrounded by 2 or more encircling membranes [42, 43], were significantly increased in Mtb-infected BMDMs at 18 h (Fig. 5G, H). However, there was no significant difference in cytosolic bacteria, which were counted as those without surrounding host membranes [42], between DMI-treated and -untreated conditions (Fig. 5H). Bacteria-containing phagosomes enclosed by a single membrane-bound organelle [42, 43] were significantly downregulated in Mtb-infected BMDMs treated with DMI (Fig. 5H). Therefore, DMI promotes the association of Mtb with autophagosomes and autolysosomes, thereby boosting phagosomal maturation during infection.

### DMI-induced autophagy is partly required for antimicrobial responses to Mtb, BCG, and Mav infection

**Fig. 6:**
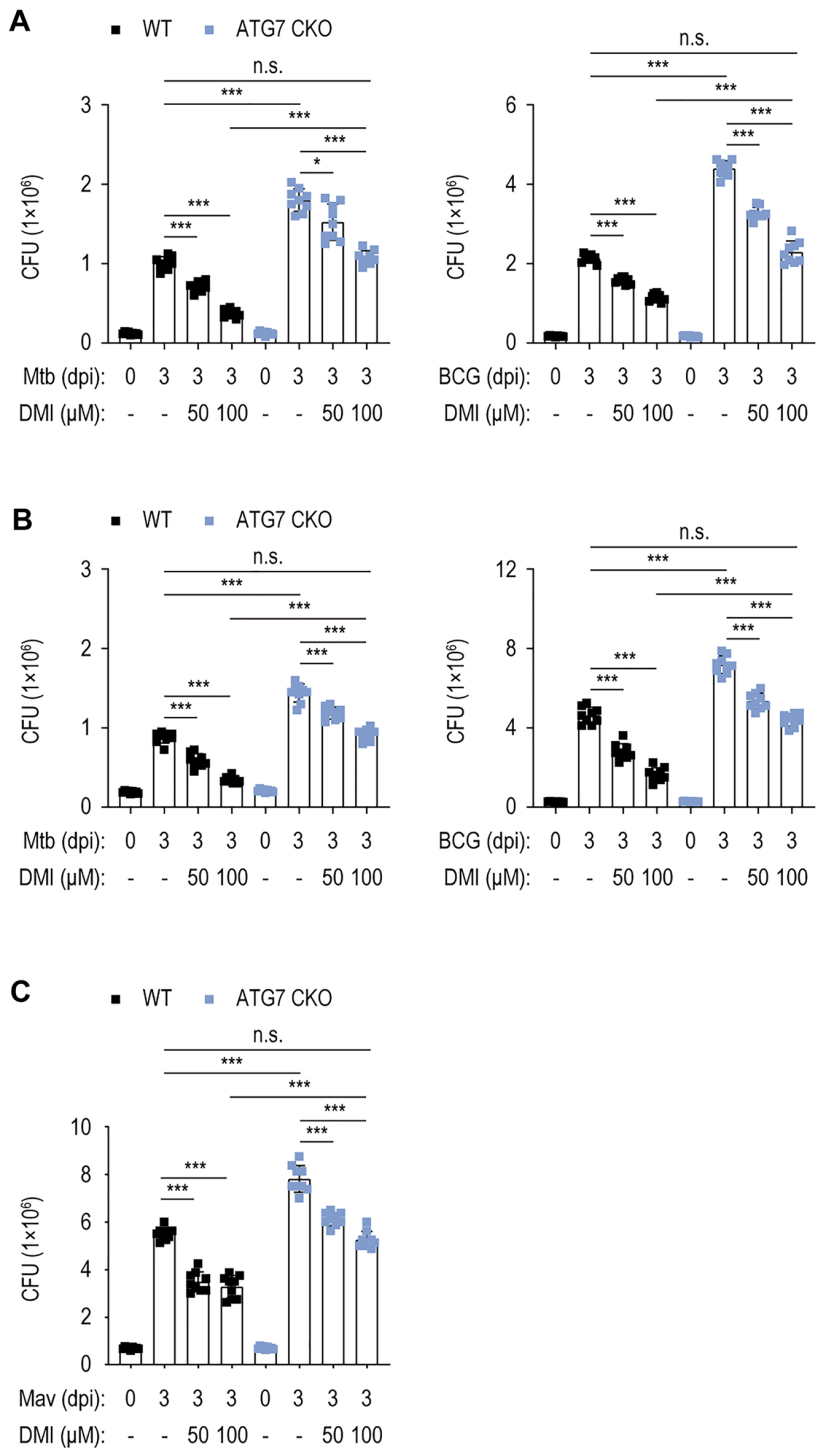
Autophagy is partially involved in the effect of DMI on antimicrobial responses in macrophages. Intracellular survival assay after Mtb, BCG, or Mav (MOI 1) in the presence or absence of DMI. **A** BMDMs from *Atg7* WT or *Atg7* cKO mice were infected with Mtb (left panel) or BCG (right panel) and treated with indicated concentration of DMI for 3 days. Cells were lysed and used to a CFU assay to examine the intracellular survival of Mtb or BCG. **B** PMs from *Atg7* WT or *Atg7* cKO mice were infected with Mtb (left panel) or BCG (right panel) and treated with indicated concentration of DMI for 3 days. Cells were lysed and used to a CFU assay to examine the intracellular survival of Mtb or BCG. **C** BMDMs from *Atg7* WT or *Atg7* cKO mice were infected with Mav (MOI 1) and treated with indicated concentration of DMI for 3 days. Cells were lysed and used to a CFU assay to examine the intracellular survival of Mav. Statistical analysis was conducted with one-way ANOVA test with Tukey’s multiple comparisons. Data shown as means ± SD from two independent experiments conducted in triplicate. *CFU* colony forming unit, *n.s.* not significant, *DMI* dimethyl itaconate. *p < 0.05 and ***p < 0.001

We next assessed whether autophagy inhibition affects DMI-mediated antimicrobial responses in BMDMs by comparing the DMI-induced intracellular survival of Mtb and BCG in BMDMs from WT mice (*Atg7*^*fl*/*fl*^) versus mice with myeloid cell-specific disruption of the crucial autophagy gene *Atg7* (*Atg7*^*fl*/*fl*^; *LysMcre*^+^; conditional homozygous knockout, cKO) (Fig. 6A). Notably, DMI significantly inhibited intracellular Mtb and BCG growth in *Atg7* WT and cKO BMDMs (Fig. 6A). However, *Atg7* cKO BMDMs showed significantly increased bacterial growth compared with *Atg7* WT BMDMs under all conditions at 3 dpi in the presence or absence of DMI. Similar pattern was observed in *Atg7* cKO PMs under all conditions in the presence or absence of DMI (Fig. 6B). Notably, the numbers of intracellular Mtb and BCG CFUs were comparable between *Atg7* cKO macrophages treated with 100 μM of DMI and SC-treated *Atg7* WT macrophages (Fig. 6A, B, for BMDMs and PMs, respectively).

DMI-mediated suppression of intracellular Mav growth was observed in *Atg7* WT and *Atg7* cKO BMDMs (Fig. 6C). The number of intracellular Mav CFUs was significantly higher in *Atg7* cKO than *Atg7* WT BMDMs in the presence or absence of DMI (Fig. 6C). As with the Mtb-infected conditions, there was no difference in intracellular Mav growth between *Atg7* cKO BMDMs treated with 100 μM of DMI and SC-treated *Atg7* WT BMDMs (Fig. 6C). Therefore, autophagy is partly required for DMI-induced antimicrobial responses in macrophages against Mtb, BCG, and Mav infections.

### DMI modulates the activation of STAT3 in mycobacterium-infected macrophages

**Fig. 7:**
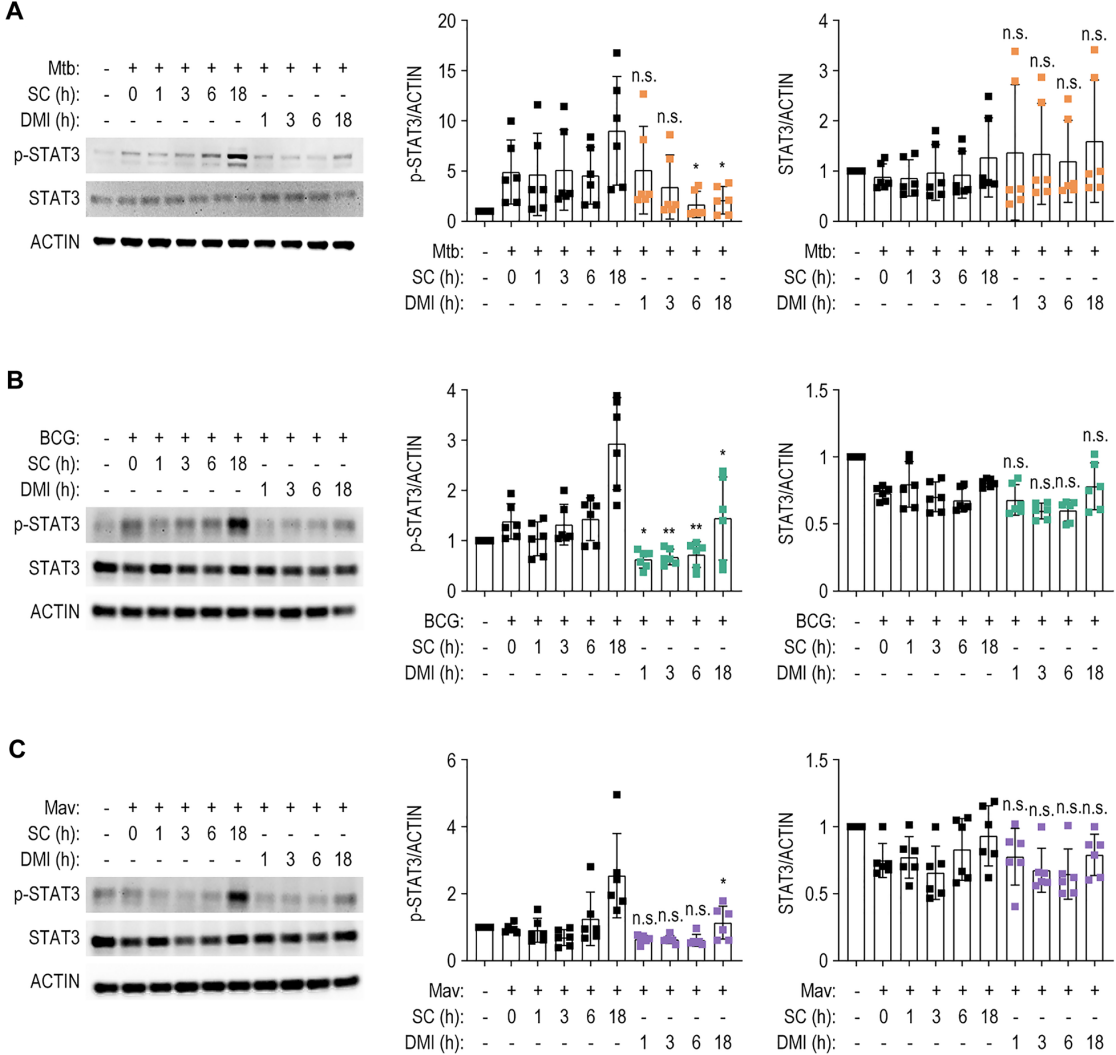
DMI treatment inhibits the activation of STAT3 in mycobacteria-infected macrophages. BMDMs were infected with Mtb (**A**), BCG (**B**), or Mav (**C**) (MOI 3) for 4 h and washed with DPBS followed by incubation with SC or DMI (100 μM) in the fresh media. The cells were harvested at the indicated times. The p-STAT3 and STAT3 levels were evaluated by Western blot analysis. Densitometry analysis of p-STAT3 and STAT3 Western blot represented in right panels. Statistical analysis was determined with unpaired Student’s *t*-test and shown as means ± SD from three independent experiments conducted in duplicate. *n.s.* not significant, *SC* solvent control, *DMI* dimethyl itaconate. *p < 0.05, **p < 0.01, and ***p < 0.001

Given that DMI suppresses IL-6 and IL-10 and induces autophagy in macrophages during infection, we evaluated its effect on the activation of the transcription factor STAT3, which modulates gene expression in response to IL-6 and IL-10 [44] and the suppression of autophagy [45, 46]. In addition, the STAT3 signaling pathway may play a detrimental role in the host defensive responses through the enhanced intracellular Mtb survival, blockade of apoptosis, and aggravation of inflammatory responses during infection and inflammation [47–49]. We thus evaluated the effect of DMI on STAT3 phosphorylation and expression. Although the activation patterns differed over time, Mtb, BCG, or Mav infection significantly increased the phosphorylation of STAT3 at Tyr-705, which was highly phosphorylated at 18 h in BMDMs (Fig. 7A–C). When BMDMs were exposed to DMI, the phosphorylated STAT3 levels were significantly downregulated at 18 h during infections of all three strains tested (Fig. 7A–C). These data suggest that DMI significantly suppresses the activation of STAT3 which signaling might be related to detrimental effects upon host responses during Mtb and NTM infections.

## Discussion

Mycobacteria–host interactions are complex and dynamic and influence the outcome of infection. Mtb and NTM have evolved multiple strategies to modulate and evade host innate signaling pathways and immune clearance [50–52]. These include interruption of phagosomal maturation [53, 54], disruption of apoptosis [55, 56] and autophagy [57], evasion of reactive oxygen species (ROS) [58, 59], deleterious type I IFN secretion [60], excessive production of inflammatory cytokines [61], and immunosuppression with increased IL-10 production [62, 63]. These events can modulate the host–pathogen relationship to favor mycobacterial survival in macrophages. Several drug candidates for HDT can reportedly overcome the immune-evasion mechanisms of each mycobacterial strain and enhance host defensive mechanisms [16, 64–70]. However, little is known about the potential drug candidate(s) that can activate innate host defense against both Mtb and NTM infections. Here, we show that DMI offers a therapeutic promise for HDT against multiple mycobacteria including Mtb, BCG, Mav, and even to MDR-Mtb. Importantly, DMI acts as a potential HDT agent through diverse mechanisms involving the maintenance of inflammatory homeostasis, enhancement of autophagy and phagosomal maturation, and suppression of STAT3 signaling.

The natural form of itaconate is produced by IRG1, which is expressed in murine and human macrophages, and *Irg1* expression is induced in host cells by Mtb infection via the Mtb ESX-1 secretion system, and the host STING, type I IFN, and TLR2-dependent signaling pathways [17, 71]. Michelucci et al. reported that IRG1 and its product itaconic acid suppress mycobacterial growth by blockade of bacterial isocitrate lyase [17]. IRG1 and its product itaconate in myeloid cells are required to control neutrophil infiltration and suppress pathological inflammation, ROS generation, and tissue injury in a mouse model of Mtb infection [19]. However, whether cell-permeable derivatives of itaconate suppress in vitro and in vivo growth of multiple mycobacteria is largely unclear. Our data is important to show that DMI has an in vivo antimicrobial activity to control bacterial growth in the lung tissues during a variety of infections caused by mycobacteria including Mtb, BCG, Mav, and even by MDR-Mtb. At least partly consistent with our findings, recent studies showed that either endogenous itaconate or itaconate derivatives potentiate intracellular killing of bacteria such as *Salmonella* Typhimurium [72, 73]. A recent study also showed that DMI administration ameliorates the cognitive deficits and proinflammatory responses in microglia caused by *Toxoplasma gondii* infection [74]. Additionally, our data partly correlated with the previous studies showing that DMI exerts anti-fungal effects in fungal keratitis [75]. Combined with our current findings, these data strongly provide a clue that DMI is being useful for the potential therapeutic modality against a variety of infection. Given that IRG1 is required to control excessive neutrophil infiltration in the lungs to ameliorate pathological inflammation and progression of Mtb infection [76, 77], further research should examine whether DMI-mediated antimicrobial defense is associated with attenuation of neutrophil recruitment in the lungs of mice infected with Mtb, BCG, Mav, or MDR-Mtb.

Although it reduced the intracellular survival of mycobacteria in macrophages, DMI did not exert a direct antimicrobial effect because a DMI concentration of 1000-fold the IC_50_ of INH is required to suppress bacterial growth under 7H9-OADC conditions. In addition, IC_50_ values of DMI showed significantly increased IC_50_ values for Mtb, BCG, and Mav, under 7H9-acetate conditions, the carbon-limiting culture conditions for activating bacterial isocitrate lyase, a target of itaconate [34], compared to those induced by INH for Mtb. Thus, antimicrobial effects of DMI are mainly mediated through multiple host-defense strategies, not by direct bactericidal activities, during mycobacterial infection. Firstly, DMI maintained the homeostasis of inflammation by reducing the level of proinflammatory (*Il6* and *Il1b*) and anti-inflammatory (*Il10*) cytokines in vivo and in macrophages during infection. Accumulating evidence suggests that DMI and OI reduce the infection-induced inflammatory responses and the release of chemokines CXCL10 and CCL2 [78]. Importantly, DMI-mediated suppression of influenza virus-induced *Cxcl10* does not depend on the expression of *Irg1* in murine macrophages [78]. Furthermore, DMI treatment alleviates the generation of inflammatory cytokines/chemokines such as IL-1β, IL-6, and IL-8 in human corneal epithelial cells through Nrf2/heme oxygenase‐1 (HO‐1) [75]. Combined with the previous findings that DMI activates the Nrf2 protein [75, 79], DMI ameliorates excessive pathologic inflammation during mycobacterial infections presumably through the activation of Nrf2, and this warrants further examination in the context of Mtb and NTM infections. Inflammatory response and production of antimicrobial factors including ROS and antimicrobial peptides are important for host immune defense against intracellular mycobacteria [80, 81]. However, the unbalanced inflammatory response to chronic infections with intracellular pathogens causes damage to the host, rendering inflammatory homeostasis as a target for HDT against Mtb and NTM, particularly drug-resistant strains [82]. Therefore, new drugs that maintain the balance between inflammatory host defense and prevention of necrotic inflammation are urgently needed. Our data revealed that DMI is a promising anti-mycobacterial therapeutic target with a balanced activity through regulating both pro- and anti-inflammatory responses during infection.

In addition, DMI enhances the *Csf2* mRNA level in the lung tissues from mice infected with Mtb and BCG, whereas it enhances *Ifng* mRNA expression in the lungs during BCG, but not Mtb, infection. In addition to IFN-γ, which is a well-known protective Th1 cytokine during Mtb infection [37, 38], emerging evidence suggests that GM-CSF is required for the restriction of Mtb infection in macrophages at least partly mediated through peroxisome proliferator-activated receptor-γ [36]. GM-CSF is mainly produced by iNKT cells and γδ T cells in early phase of infection [36] and functions as an antibacterial effector cytokine participating in IFN-γ-independent host protection against Mtb infection [35]. Thus, the DMI effects may contribute to improve host defense through keeping immune homeostasis and activating protective cytokine generation, during Mtb and NTM infections.

Autophagy, a cell-autonomous defense mechanism, is a possible target of HDT against mycobacterial infection [69, 70, 83]. Several autophagy-activating factors enhance innate host defense and lysosomal degradation of intracellular mycobacteria [67–70, 83]. DMI robustly activated autophagy, which partly affects intracellular mycobacterial survival in *Atg7* cKO macrophages. These data suggest that DMI-mediated autophagy activation is necessary but not sufficient, to suppress intracellular mycobacterial survival. DMI further enhances the colocalization of Mtb-ERFP with autolysosomes, indicating that DMI-induced autophagy may promote phagosomal maturation against Mtb infection. These results are in partial agreement with a recent report that OI enhances autophagy in chondrocytes by suppressing PI3K/Akt/mTOR signaling [84]. However, OI also suppresses autophagy and ROS generation to exert an anti-fibrotic effect in renal tissue [85]. These data suggest that cell-permeable itaconate modulates host autophagy in a context-dependent manner.

Importantly, DMI significantly reduced the STAT3 phosphorylation levels in macrophages against Mtb, BCG, or Mav infection. STAT3 is a transcription factor activated by cytokines such as IL-6, IL-10, and growth factors, and is implicated in the activation of Th17 cell responses and autoimmune diseases as well as anti-inflammatory responses [44, 86, 87]. In mycobacterial infection, the role of STAT3 signaling in the immune response has been debated [88]. In human innate immune responses, STAT3 signaling and TLR4 pathway activation are important in the vitamin D-mediated antimicrobial pathways in macrophages [89], although the molecular mechanisms are unknown. However, the p-STAT3 inhibitor AG-490 protected against lung injury in a mouse model of type 2 diabetes-associated TB [47]. The inhibition of p-STAT3 by AG-490 improves mouse survival and histopathological findings and ameliorates inflammation, fibrosis, and Mtb growth [47]. Therefore, DMI-mediated STAT3 inhibition is likely responsible for the suppression of pathological inflammation during mycobacterial infection. Also, STAT3 signaling activation is associated with anti-apoptotic responses, favoring the intracellular survival of Mtb [48]. In addition, Mtb Rv2145c-mediated intracellular bacterial growth is dependent on STAT3-mediated IL-10 production [49], suggesting an immunosuppressive role for STAT3. Indeed, STAT3 signaling suppresses autophagy by multiple molecular mechanisms including transcriptional regulation of autophagy-related genes and IL-10-mediated inhibition of autophagy [45, 46]. Together with its suppressive functions upon autophagy pathway, the STAT3 pathway contributes to intracellular Mav survival in macrophages [90]. Further research is needed to determine whether DMI-mediated suppression of STAT3 signaling underlies the activation of autophagy induced by DMI treatment.

## Conclusions

In summary, the present study has demonstrated that DMI shows potent antimicrobial activities during Mtb, BCG, Mav, or even to MDR-Mtb infection. In addition, DMI functions as an important regulator of inflammatory homeostasis, activation of autophagy, and control of STAT3 signaling. DMI-induced autophagy partly contributes to improve host defenses against mycobacterial infections. Together, we propose DMI as a new promising candidate for HDT against both Mtb and NTM infections through orchestrating multiple innate immune strategies.

## Supplementary Information

**Additional file 1: Figure S1.** Direct effect of DMI on various mycobacteria under 7H9-OADC culture conditions. **Figure S2.** Direct effect of DMI on various mycobacteria under carbon-limiting conditions. **Figure S3.** The treatment with DMI regulates the expression level of proinflammatory cytokines in both Mtb- and Mav-infected murine macrophages.

## Data Availability

All data generated or analyzed during this study are included in this published article and its Additional files.

## Abbreviations

Baf-A1: Bafilomycin A1
BCG: *Mycobacterium bovis* Bacillus Calmette–Guérin
BMDM: Bone marrow-derived macrophage
CFU: Colony-forming unit
cKO: Conditional knockout
DAPI: 4′-6-Diamidino-2-phenylindole
DMEM: Dulbecco’s modified Eagle’s medium
DMI: Dimethyl itaconate
DPBS: Dulbecco’s Phosphate-Buffered Saline
DPBS-T: Dulbecco’s Phosphate-Buffered Saline added with 0.05% Tween-80
Dpi: Days post infection
EGFP: Enhanced green fluorescent protein
ELISA: Enzyme-linked immunosorbent assay
ERFP: Expressing red fluorescent protein
GM-CSF: Granulocyte-macrophage colony-stimulating factor
HDT: Host-directed therapeutics
H&E: Hematoxylin and eosin
HO-1: Heme oxygenase‐1
IC50: Half maximal inhibitory concentration
IFN: Interferon
IL: Interleukin
INH: Isoniazid
IRG1: Immune-responsive gene 1
LAMP1: Lysosomal-associated membrane protein 1
LC3: Microtubule-associated protein 1 light chain 3 β
LPS: Lipopolysaccharide
Mav: *M. avium*
MDR: Multidrug-resistant
MOI: Multiplicity of infection
Mtb: *M. tuberculosis*
Nrf2: NF-E2 p45-related factor 2
NTM: Nontuberculous mycobacteria
OADC: Oleic albumin dextrose catalase
OI: 4-Octyl itaconate
PBS: Phosphate-buffered saline
PM: Peritoneal macrophage
p-STAT3: Phospho-signal transducer and activator of transcription 3
qRT-PCR: Quantitative real-time polymerase chain reaction
ROS: Reactive oxygen species
SC: Solvent control
SD: Standard deviation of the mean
SEM: Standard error of the mean
STAT3: Signal transducer and activator of transcription 3
TB: Tuberculosis
TEM: Transmission electron microscopy
TNF: Tumor necrosis factor
WT: Wild-type
