# Supplementary material for: Dimethyl itaconate is effective in host-directed antimicrobial responses against mycobacterial infections through multifaceted innate immune pathways
Source: Cell Biosci. 2023 Mar 8;13:49. doi: 10.1186/s13578-023-00992-x (PMC9993662; doi:10.1186/s13578-023-00992-x)
Supplement: Supplementary file 1 — Additional file 1: Figure S1. Direct effect of DMI on various mycobacteria under 7H9-OADC culture conditions. Figure S2. Direct effect of DMI on various mycobacteria under carbon-limiting conditions. Figure S3. The treatment with DMI regulates the expression level of proinflammatory cytokines in both Mtb- and Mav-infected murine macrophages. [file 13578_2023_992_MOESM1_ESM.docx]

Additional file 1 for

**Dimethyl itaconate is effective in host-directed antimicrobial responses against mycobacterial infections through multifaceted innate immune pathways**

Young Jae Kim^a,b,c,d†^, Eun-Jin Park^a,b†^, Sang-Hee Lee^e^, Prashanta Silwal^a,b^, Jin Kyung Kim^f^, Jeong Seong Yang^g^, Jake Whang^g^, Ji-Chan Jang^h^, Jin-Man Kim^b,c,i^, and Eun-Kyeong Jo^a,b,c^*

***Correspondence**

Eun-Kyeong Jo (hayoungj@cnu.ac.kr)

Department of Microbiology, Chungnam National University School of Medicine, 266 Munhwa-ro, Jung-gu, Daejeon, 35015, South Korea.

**Contents of Additional file 1:**

Fig. S1 Direct effect of DMI on various mycobacteria under 7H9-OADC culture conditions.

Fig. S2 Direct effect of DMI on various mycobacteria under carbon-limiting conditions.

Fig. S3 The treatment with DMI regulates the expression level of proinflammatory cytokines in both Mtb- and Mav-infected murine macrophages.

**
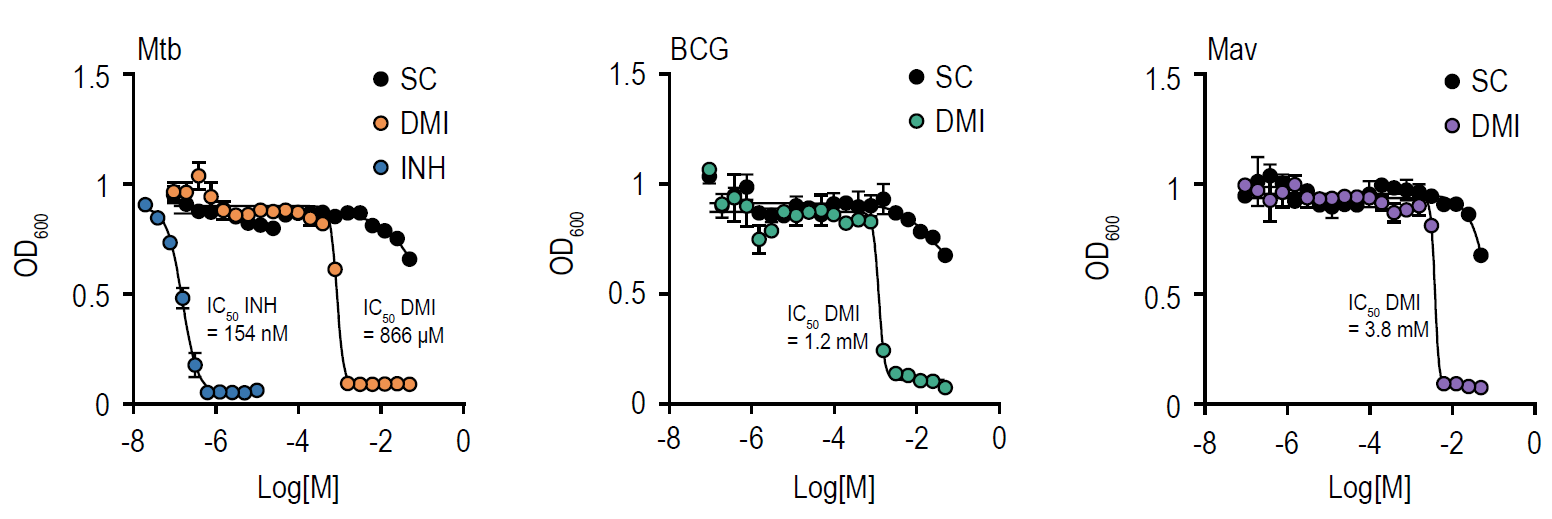
**

**Fig. S1. Direct effect of DMI on various mycobacteria.** The effect of DMI was examined on Mtb, BCG, or Mav cultures. Dose-response curves were depicted from the OD600 of test plates after incubation of appropriate periods at 37℃. The experiments were carried out with three biological replicates and presented as the mean value ± SEM for each concentration point. This graph was plotted from a representative experiment. SC, solvent control; DMI, dimethyl itaconate; INH, isoniazid; OD, optical density; IC_50_, half-maximal inhibitory concentration.

**
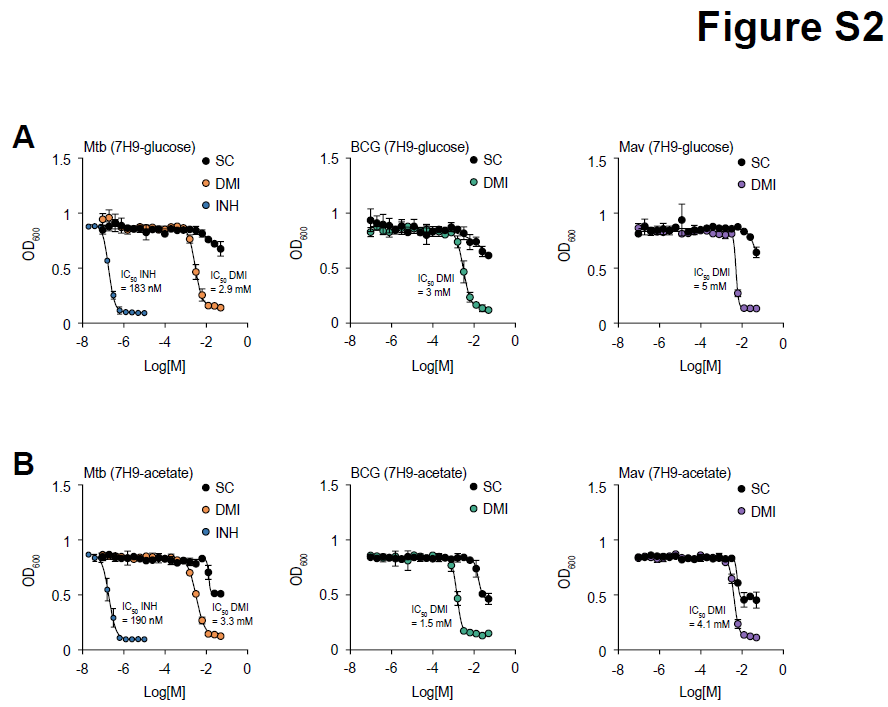
**

**Fig. S2. Direct effect of DMI on various mycobacteria cultured under carbon-limiting conditions.** The effect of DMI was examined on Mtb, BCG, or Mav cultures. Dose-response curves were depicted from the OD600 of test plates prepared with 7H9-glucose (A) and 7H9-acetate (B) after incubation of appropriate periods at 37℃. The experiments were carried out with three biological replicates and presented as the mean value ± SD for each concentration point. This graph was plotted from a representative experiment. SC, solvent control; DMI, dimethyl itaconate; INH, isoniazid; OD, optical density; IC_50_, half-maximal inhibitory concentration.

**
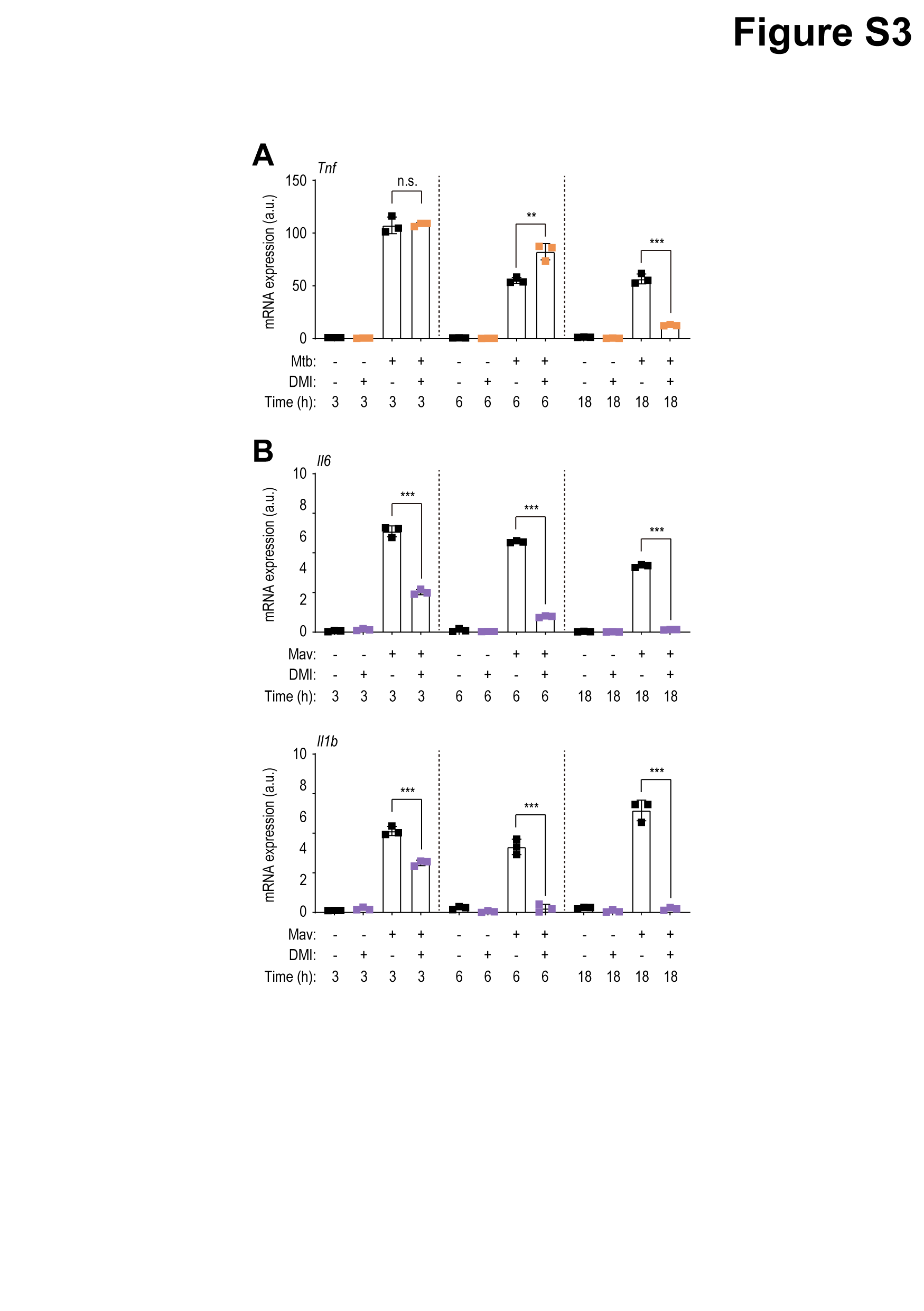
**

**Fig. S3. The treatment with DMI regulates the expression level of proinflammatory cytokines in both Mtb- and Mav-infected murine macrophages.** Mtb- **(A)** or Mav- **(B)** (MOI 3) infected BMDMs for 4 h were incubated in the freshly changed media treated with SC or 100 μM of DMI. The cells were lysed at the indicated times (3, 6, or 18 h) and used to qRT-PCR analysis to estimate the expression level of **(A)** *Tnf*, **(B)** *Il6,* and *Il1b*. Statistical analysis was conducted with unpaired Student’s *t*-test and shown as means ±SD from two independent experiments conducted in triplicate. The results were shown as representative experiments. DMI, dimethyl itaconate; n.s., not significant; a.u., arbitrary unit. ***p* <0.01, ****p* <0.001 and *****p* <0.0001.
